# Phyllodes Tumor of the Breast: 307 Treated Cases, the Largest Mexican Experience at a Single Breast Disease Institution

**Published:** 2016

**Authors:** Eva Ruvalcaba-Limón, Josefina Jiménez-López, Verónica Bautista-Piña, Julio Ramírez-Bollas, Flavia Morales-Vásquez, Carlos Domínguez-Reyes, Antonio Maffuz-Aziz, Sergio Rodríguez-Cuevas

**Affiliations:** 1 *Dept. of Breast Surgical Oncology, * *Instituto de Enfermedades de la Mama y Fundación del Cáncer de Mama (IEM-FUCAM), Mexico City, Mexico*; 2 *Dept. of Pathology, * *IEM-FUCAM, Mexico City, Mexico*; 3 *Dept. of Medical Oncology, * *IEM-FUCAM, Mexico City, Mexico*

**Keywords:** Breast tumors, Fibroepithelial neoplasms, Phyllodes tumor

## Abstract

**Background:**

Phyllodes tumor (PT) of the breast in Hispanic patients is more frequently reported with large tumors and with more borderline/malignant subtypes compared with other populations. The objective of this study was to describe characteristics of patients with PT and to identify differences among subtypes in a Mexican population.

**Methods:**

A retrospective study was conducted on patients with PT. Sociodemographic, histopathologic, and treatment characteristics were compared among subtypes, including only surgically treated cases due the complete surgical-specimen study requirement for appropriate WHO classification.

**Results:**

During 10 years, 346 PT were diagnosed; only 307 were included (305 patients), with a mean age of 41.7 yr. Self-detected lump took place in 91.8%, usually discovered 6 months previously, with median tumor size of 4.5 cm. Local wide excisions were done in 213 (69.8%) and mastectomies in 92 (30.1%). Immediate breast reconstruction took place in 38% and oncoplastic procedures in 23%. PT were classified as benign in 222 (72.3%) cases, borderline in 50 (16.2%), and malignant in 35 (11.4%), with pathological tumor size of 4.2, 5.4, and 8.7 cm, respectively (*P*<0.001). Patients with malignant PT were older (48 yr), with more diabetics (14.3%), less breastfeeding (37.1%), more smokers (17.1%), with more postmenopausal cases (42.9%), and older age at menopause (51.5 years) compared with the remaining subtypes (*P*<0.05). Relapse occurred in 8.2% of patients with follow-up.

**Conclusion:**

In comparison with other Hispanic publications, these Mexican patients had similar age, with smaller tumors, modestly higher benign PT, fewer malignant PT, and lower documented relapse cases.

## Introduction

“Phyllodes tumor (PT) of the breast is a rare fibroepithelial neoplasm that accounts for <1% of all breast tumors” (1). Very large size or rapid growth may suggest PT rather than fibroadenoma. Large tumors produce skin ulceration or chest wall invasion indistinguishable from breast cancer. The first description of PT was presented by Müller in 1838 (2); since that time, PT has acquired >60 different synonyms (3). The most common classification was proposed by Pietruszka and Barnes (4), improved by Azzopardi (5) and Salvatori (6), and adapted by WHO (7); it divides PT into benign, borderline, or malignant PT. Classification may be problematic because of the presence of features from >1 category, cystic areas, and necrosis. Definitive diagnosis is determined with the entire surgical specimen study. Fine needle aspiration is the less accurate procedure, while Core-needle biopsy (CNB) entertains high positive and negative predictive values; morphologic variability by itself compromised accurate classification sampled by these methods (8). Average ages at presentation are 39.4 to 47 yr, and mean tumor size is 5 to 8.3 cm (8-14). Hispanic women appear to have larger tumors and aggressive subtypes compared with non-Hispanic cases (15).

Surgical treatment is the cornerstone of PT management, wide local excision with at least a 1 cm three-dimensional margin is the main option, and large tumors could require mastectomy. Axillary metastases are observed in <5% of cases and axillary surgery is not indicated unless worrisome nodes are clinically evident. Margin status and tumor size comprise the most important predictive factors of local recurrence and distant metastases (6, 11, 13). Borderline and malignant PT have a high risk of recurrence, and mastectomy is recommended (14, 16). 

The objective of this study was to describe the clinical, histopathological, and therapeutic aspects of PT of the breast in a sample of Mexican feminine population and to compare our results with those of other publications that included Hispanic patients. 

## Materials and Methods

After approval by the Institutional Review Board of Instituto de Enfermedades de la Mama – FUCAM (IEM-FUCAM), a retrospective cross-sectional study was conducted of consecutive patients with PT between August 2005 and August 2015. IEM-FUCAM is a non-profit and high specialty hospital that attends to open population of Mexico City’s Federal District and Metropolitan Area. Inclusion criteria included the following: female patients ≥16 yr of age with a diagnosis of PT in breast tissue of CNB or in definitive surgical specimen. The type of surgery was performed according to the medical staff clinical evaluation consensus, and axillary lymph node dissection was performed in patients with clinical suspicion of lymph node metastases. The variables studied involved sociodemographic and gynecologic aspects, tumor features, treatment, and relapse if this occurred. PT classification encompasses criteria adopted by the WHO Working Group (7,17,18), which evaluate stromal hypercellularity, cellular pleomorphism, mitoses, margins, stromal pattern, and heterologous elements and which divides tumors into benign, borderline, and malignant PT. 

Data were obtained from the patients’ clinical files and from histopathological reports of their tumor tissues. Descriptive statistical techniques were used to evaluate frequency distribution. To distinguish differences among the three subtypes, Pathologists need to sample all surgical specimens in order to classify the latter, including, only for this purpose, surgically treated cases. 

Differences among continuous variables were tested employing Analysis of variance (ANOVA) for normal distribution data or Kruskal-Wallis H-test, and differences in distribution of categorical variables were analyzed employing the Pearson chi-square test or Fisher exact test. Outcome was analyzed only in patients with verifiable follow-up. All *P-*values ≤0.05 were considered statistically significant, and all *P*-values were two-sided. Statistical analyses were performed with the SPSS software package (Chicago, IL, USA) version 16.0 for Windows.

## Results

**General Population**

During 10 yr, IEM-FUCAM registered 346 PT in 344 patients; 39 (11.3%) cases were excluded because patients decided to undergo surgery at another hospital due their medical affiliation with other institutions. The present study included 305 patients with 307 PT, two (0.6%) cases with multifocal PT. Mean age was 41.7 yr; co-morbid states were present in <13% (Table 1). Mean Body mass index (BMI) was 27.0 kg/m^2^ with 202 (66.2%) patients in the overweight/obese range. Premenopausal status predominated (77.7%). Age at menopause was 46.2 yr in the 68 postmenopausal patients, and use of hormonal replacement therapy was rare. 

**Table 1.** Characteristics of 305 patients with phyllodes tumor

Variable	Value
Age (years)	42 (16-75)
Systemic high blood pressure	38 (12.4%)
Diabetes mellitus	18 (5.9%)
Smoking	21 (6.8%)
BMI (kg/m²)	27.0±5.42
Normal (18.5-24.99 kg/m²)	103 (33.7%)
Overweight or obesity (BMI ≥25 kg/m^2^)	202 (66.2%)
Age at menarche (years)	12.72±0.6
Age at first delivery (years)	22.54±5.03
Number of pregnancies	2 (0–10)
Breastfeeding (positive)	192 (62.9%)
Use of hormonal contraceptives	79 (25.9%)
Premenopausal	237 (77.7%)
Postmenopausal	68 (22.2%)
Age at menopause (yr) n=68	46.25±6.47
Use of hormonal replacement therapy, n=68	3 (4.4%)

Nominal variables are expressed as number and percentage. Scale variables are expressed as mean±SD or median with minimal-maximal values. BMI=Body mass index

Self-detected palpable lump was the most frequent condition (92.4%), usually discovered 6 months previously, with a median tumor size of 4.5 cm. Non-palpable or not-well-identified tumor during physical examination occurred in 25 cases (8.1%), all of these asymptomatic. Mammary glands were similar affected, and external quadrants were involved in 188 (61.2%) cases, followed by central tumors (Table 2). 

**Table 2.** Characteristics of 307 phyllodes tumors

Variable	Value
Clinical manifestations	
Self-detected lump	282 (91.8%)
Asymptomatic	25 (8.1%)
Disease evolution time in symptomatic cases (months) n=282	6 (1–96)
Right mammary gland	154 (50.1%)
Left mammary gland	153 (49.8%)
Mammary quadrant affected	
External quadrants	188 (61.2%)
Internal quadrants	52 (16.9%)
Central	67 (21.8%)
Type of biopsy	
Core-needle biopsy in palpable lump	281 (91.5%)
Core-needle biopsy (ultrasound-guided)	21 (6.8%)
Excisional biopsy	5 (1.6%)
Histopathology	
Benign	222 (72.3%)
Borderline	50 (16.2%)
Malignant	35 (11.4%)
Final surgical procedure	
Local wide excision	213 (69.8%)
Mastectomy*	92 (30.1%)
Axillary lymph node dissection^†^	5 (1.6%)
Immediate breast reconstruction in mastectomy cases, n=92	35 (38.0%)
Oncoplastic surgery in local wide excision, n=213	49 (23.0%)
Tumor size^‡^	
Clinical tumor size (palpable) n=282	4.5 (1–39)
Mammography tumor size, n=218	4.0 (1–25)
Ultrasound tumor size, n=290	3.9 (0.6–32)
Pathologic tumor size, n=307	4.8 (1–30)

Nominal variables are expressed as number and percentage. Scale variables are expressed as mean±SD or median with minimal-maximal values.

BMI=Body mass index.

* Specimens from two mastectomies with multifocal disease; ^†^Patients with clinical suspicion of lymph node metastases during the surgical procedure; ^‡^Maximal diameter in centimeters.

All cases with the exception of five underwent preoperative biopsy; 281 (91.5%) tumors underwent CNB in a Physician´s office, and 21 (6.8%) were by Ultrasound-guided CNB. Not all biopsies were reported as PT; 64 (21.1%) were reported as fibroadenoma; among these, 59 (92.1%) were benign PT in the surgical specimen and five (7.8%), borderline. The five cases with excisional biopsy without preoperative biopsy were performed because Ultrasound (US) images were compatible with fibroadenoma (three cases), intracystic papilloma (one case), and multifocal PT (one case). 

Surgical treatments were conducted in 305 patients. Second surgeries were performed in 39 (12.7%) patients due to close or positive margin status on first surgery (31 re-excisions and eight mastectomies). Re-excision procedures were performed in cases with initial diagnosis of fibroadenoma in biopsy reports (53.8%) and in PT with margins <5 mm (46.2%). Final surgical procedures included 213 (69.8%) local wide excisions and 92 (30.1%) mastectomies. Two patients with total mastectomy had multifocal disease (benign/benign and borderline/benign PT). Axillary lymph node dissection was performed in five cases of mastectomy due to suspicious lymph nodes during surgery, without lymph node metastases in the histopathological study of these patients. No sentinel lymph node biopsies were done. Median tumor size in surgical specimen was 4.8 cm, with median tumor size of 3.7 and 10 cm in local wide excisions and mastectomies, respectively. Immediate breast reconstruction in mastectomies took place in 35 (38%) cases, and oncoplastic surgery in 49 (23%) patients who had undergone wide excision. 

**Differences between the three subtypes**

After complete histopathological study of the 307 specimens, PT were classified as benign in 222 (72.3%) cases, borderline in 50 (16.2%), and malignant in 35 (11.4%) (Table 3). Patients with benign PT were more often premenopausal (82.1 vs. 57.1-62.0%; *P<*0.001), and with less diabetes (4.1 vs. 8.0-4.3%; *P*=0.044). Patients with malignant PT were older (48 years), with more cases of diabetes mellitus (14.3%), less breastfeeding (37.14%), more smokers (17.1%), with more postmenopausal cases (42.9%), and older age at menopause (51.5 years) compared with the remaining subtypes, all with statistically significant differences. These patients had more systemic high blood pressure cases compared to other PT subtypes, but without a statistically difference.

**Table 3:** Sociodemographic, clinical, and treatment differences among phyllodes tumor subtypes

Variable	Benign ***n=*222**	Borderline *** n=*50**	Malignant *** n=*35**	***P*** **-value**	
Age (yr)	41 (16-75)	46.5 (22-68)	48 (23-69)	<0.001
Systemic high blood pressure	23 (10.4%)	7 (14.0%)	8 (22.9%)	0.105
Diabetes mellitus	9 (4.1%)	4 (8.0%)	5 (14.3%)	0.044
Smoking	8 (3.6%)	7 (14.0%)	6 (17.1%)	0.001
BMI (kg/m²)	26.8±5.6	27.1±4.7	28.4±4.9	0.082
Age at menarche (years)	12.7±1.6	12.5±1.5	12.8±1.1	0.675
Age at first delivery (years)	22.2±4.7	23.6±6.1	23.1±5.1	0.595
Number of pregnancies	2 (0–10)	2 (0–9)	2 (0–5)	0.181
Breastfeeding (positive)	144 (64.8%)	35 (70.0%)	13 (37.1%)	0.002
Use of hormonal contraceptives	55 (24.7%)	14 (28.0%)	10 (28.5%)	0.806
Premenopausal	188 (84.7%)	31 (62.0%)	20 (57.1%)	<0.001
Postmenopausal	34 (15.3%)	19 (38.0%)	15 (42.9%)	
Use of hormonal replacement therapy, n=68	1 (2.9%)	2 (10.0%)	0	0.304
Age at menopause (years), n=68	44.4±5.4	45.6±6.1	51.5±5.2	0.001
Clinical manifestations				
Self-detected lump	200 (90.1%)	47 (94.0%)	35 (100%)	0.114
Asymptomatic	22 (9.9%)	3 (6.0%)	0	
Disease evolution time in symptomatic cases (months) n=282	6.5 (1-96)	7 (1-180)	5 (1-96)	0.379
Mammary gland affected				
Right mammary gland	113 (50.9%)	26 (52.0%)	15 (42.8%)	0.649
Left mammary gland	109 (49.1%)	24 (48.0%)	20 (57.1%)	
Clinical tumor size*	4.2 (1-21)	7.0 (1.4-30)	8.0 (3-39)	<0.001
Pathological tumor size*	4.2 (1-22)	5.4 (2-18.3)	8.7 (3.1-30)	<0.001
Final surgical procedures, n=305	221	49	35	
Wide excision, n=213	177 (80.1%)	30 (61.2%)	6 (17.1%)	<0.001
Mastectomy†, n=92	44 (19.9%)	19 (38.7%)	29 (82.8%)	
Immediate breast reconstruction in mastectomy	15 (34.1%)	9 (47.3%)	11 (37.9%)	<0.001
Oncoplastic surgery in local wide excision	37 (20.9%)	8 (26.6%)	2 (33.3%)	<0.001
Tumor size in mastectomy specimen^†^	10.4 (3.2–22)	9.5 (2.7-18.3)	9.3 (4.5–30)	
Tumor size in local wide excision specimen	3.5 (1–11)	4.6 (2–12.5)	4.2 (3.1–13)	

Nominal variables are expressed as number and percentage. Scale variables are expressed as median with minimal-maximal values.

BMI=Body mass index; cm=centimeters.

* Maximal tumor diameter in centimeters;

† Specimens from two mastectomies with multifocal disease both performed due to huge benign phyllodes tumor.

Age at menopause increased according to subtype severity. All patients with malignant PT had a self-detected, huge, and rapid growth lump, quite more frequently in the left breast. Clinical (8 cm) and pathological (8.7 cm) tumor sizes were statistically significantly larger compared with the other subtypes, and mastectomies were more frequent (82.8%). 

Median pathological tumor size was progressive according to histological subtype: 4.2 cm in benign; 5.4 cm in borderline, and 8.7 cm in malignant PT, this latter variable with a statistically significant difference. Fifty-three patients with tumors >5 cm could be treated with conservative surgery due to big mammary gland sizes with acceptable cosmetic results. The largest tumors treated at the Institute included three malignant 22.5, 24 and 30 cm tumors, and a 22 cm benign PT. Immediate breast reconstruction was performed more often in borderline PT cases (47.3 vs. 34.0-37.9%; *P*<0.001). 

Preoperative histopathological diagnosis by CNB was different compared to complete surgical specimen in 70 (23.1%) cases (Table 4). Fibroadenoma were diagnosed in 59 (27%) benign PT, and in five (14%), borderline PT. There was less discordant biopsy diagnosis in malignant PT.

**Table 4:** Histopathological diagnosis in core-needle biopsy and in surgical specimen

		**Surgical specimen diagnosis**			
Preoperative diagnosis by biopsy	Benign *n=*222		Borderline * n=*50	Malignant * n=*35	
No biopsy, n=5	4		1	-	
Core-needle biopsy, n=302	218		49	35	
Fibroadenoma, n=64	59 (27.0%)		5 (14.0%)	-	
PASH, n=6	5 (2.3%)		1 (2.0%)	-	
Non-specific fibroepithelial neoplasia, n=39	30 (13.7%)		3 (14.0%)	6 (17.1%)	
Phyllodes tumor, n=193	124 (56.8%)		40 (81.6%)	29 (82.8%)	

PASH=Pseundoangiomatous stromal hyperplasia of the breast.

Fifty one patients continue their follow-up at hospitals closer to their residence, the majority of them in other cities or states in Mexico, without knowledge of their condition due to lack of access to patients’ information or changes in address. For the outcome analysis, these patients were considered as missing cases, and included only 254 patients with verifiable follow-up. After a mean follow-up of 36.2 months, corroborated recurrence occurred in 21 cases (8.2%) of the 254 patients. Of these 21 cases, only nine patients developed recurrence during follow up at our Institute; the remaining 12 cases developed local relapse prior to their first visit to our Institution; the tumor of these 12 patients were surgically treated with enucleation in another hospital, with at least one positive margin in the surgical specimen. Median relapse time to first recurrence in all 21 cases with recurrence was 6 months (range, 1-72 months), earlier in malignant PT compared with benign PT (Table 5). Seven patients had a second recurrence, and one patient, a third recurrence. Malignant PT developed more recurrences compared with benign/borderline subtypes (18.7 vs. 6.7-6.9%, *P*<0.001). Local recurrence in the same mammary gland was the most frequent site of relapse in benign and borderline PT, while systemic recurrence (lung and bone) was the most common in malignant PT. Two cases with malignant PT and pulmonary recurrence died due to this disease; one of these two cases had simultaneous lung and axillary lymph node recurrence. Adjuvant radiotherapy was administered to 18 patients, nine (50%) in the recurrent setting and nine (50%), due to malignant PT with huge tumors, close margins, and/or skin or muscle affection.

**Table 5:** Characteristics of recurrence

	Benign PT *n=*179	Borderline PT * n=*43	Malignant PT * n=*32	*p*-value
Follow-up time (months)	37.7 (1-113)	32.7 (1-118)	30.6 (1-105)	0.161
Relapse time of first recurrence (months)	12 (6-72)	7 (3-24)	5 (1-60)	0.202
Patients with relapse, n=21	12 (6.7%)	3 (6.9%)	6 (18.7%)	<0.001
Site of first relapse				
Local	12 (100%)	3 (100%)	1 (16.6%)	
Lung	-	-	3 (50.0%)*	
Bone	-	-	2 (33.3%)	

PT=Phyllodes tumor.

* One case of malignant PT had simultaneous lung and axillary lymph node recurrence.

**Table 6:** Published experience of phyllodes tumor in Latin America

Reference		Number of cases	Age*	Tumor size†	Benign (%)	Borderline (%)	Malignant (%)
Rodríguez et al. (10) Colombia 1994	146 (in 10y)		44	7.6 (2–40)	69.8	13.6	16.4
Meneses et al. (14)/ México 2000	45 (in 12y)		44	NA (5–18)	49.0	18.0	33.0
Rodríguez et al. (9)/Venezuela 2003	55 (in 16y)		39	NA (1.5–60)	63.6	NA	36.3
Pérez et al. (21)/ Chile 2007	39 (in 21y)		44	8.3 (2–28)	82.0	7.6	10.2
Aranda et al. (22)/ México 2009	16 (in 11y)		35	6.1 (1.3-15)	87.5	0	12.5
Ibáñez et al. (23)/ Chile 2010	11 (in 8y)		42	NA (2.3–18)	60.0	20.0	20.0
Pimiento et al.‡ (15)/ USA 2011	53 (in 11y)		44	6.6	30.0§	48.0§	21.0§
Velázquez et al. (24)/ México 2013	22 (in 13y)		42.0	7.4	59.1	36.4	4.5
Present study/ México	307 (in 10y)		42.0	4.5 (1–39)	72.3	16.2	11.4

y=years; NA=Not available.

* Age in years, mean or median;

† Tumor size in centimeters, mean, or median; ^‡^Including also non-Hispanic patients;

§ Approximately.

## Discussion

PT is an uncommon biphasic neoplasm of the breast (1). Large series have accumulated information for many years, such as Barrio et al. in the U.S. with 293 cases in 51 years (19), or in Singapore with 605 patients during the authors’ 18-year study (20). Some Latin-American institutions have reported their experience, usually with few patients over a long time (Table 6). At IEM-FUCAM, during a 10-year period, we documented 346 PT and 4,198 cases of breast cancers. During this period, 307 cases of PT were treated at our Institute.

According to the WHO classification (4, 5, 17), benign, borderline, and malignant PT have been reported at 34.5 to 74.6%, 13.6 to 39.5%, and 16.43 to 50%, respectively (6,9-12,14,25). In our surgically treated cases, benign PT (72.3%) was not much higher than that reported by other authors; borderline PT was similar (16.2%), and malignant PT was lower (11.4%). PT usually appears in women aged 40 yr with an average age between 35 and 47 yr (13, 22); our study fell within the published range. Very young patients have been reported with PT: at 9 yr (6) in Italy, and at 11 yr. (26) in Argentina, as well as in older patients, such as women aged 77, 84, and 88 yr in Venezuela, Colombia, the U.S., and Italy (6,9,10,27). The age range in the present study was 16 to 75 yr. Age increases with subtype severity (11), finding that this was repeated in our study. 

Co-morbidities are rarely reported. In the present study, prevalence of co-morbidities was not very low. Patients with malignant subtype had more diabetes mellitus, more systemic arterial hypertension, there were and more smokers. BMI range was similar that of the Aranda et al. (22) series, which reported 19.9 to 39.6 kg/m², and that in the present study was 18.5 to 54.7 kg/m² with 66.2% within the overweight/obesity range, similar to general feminine population in Mexico. Patients with malignant PT had the highest BMI (28.4 kg/m^2^) compared with other subtypes. Premenopausal status is reported as between 64 and 74.2% (9,27), similar to our findings (77.7%), being more prevalent in patients with benign PT (84.7%; *P*<0.001) and with more postmenopausal patients in malignant PT (42.9%;* P*<0.001). Age at menopause was 46.2 yr, with ascending age in subtype severity (44.4, 45.6, and 51.5 yr). Cases with malignant PT had less breastfeeding (37.1%) compared with other subtypes. The majority of other hormonal aspects did not show any difference.

Average tumor size is variable (5.0-8.3 cm), with a wide range (0.9-25.0 cm) (8,9,11−14). In the present study, tumor size range was between 1 and 39 cm, and median clinical tumor size was 4.5 cm, smaller than in other Hispanic populations; pathological tumor size were reported as 4.8 cm and within a range of 1 to 30 cm in surgical specimens, similar to that in other series. One of the largest PT was a case with 60 cm malignant PT (9) in Venezuela. In another Mexican series (14), tumor size for benign, borderline, and malignant PT was 12.9, 8.0, and 10.3 cm, respectively, larger than our histopathological measurements (4.2, 5.4, and 8.7 cm, respectively). 

Self-detection of a lump was the most common symptom (91.8%) with a 6-month disease evolution time, very similar to those of other authors who reported self-detection of 60 to 100% at a similar evolution time (9−11,22,27). Tumors <3 cm are rarely diagnosed. In the 6,923 vacuum-assisted breast biopsies performed in Korea, of the 53 PT identified, 25.8% were non-palpable lumps (28). In a similar study (8) of 2,866 CNB carried out, 25 lesions corresponded to PT. In places with breast cancer screening programs, tumors could be detected when they were smaller, and asymptomatic PT could be as high as 31% (8,15). In Hispanic patients, asymptomatic PT is lower (2.8-5.1%) (9,21). Ibáñez et al. (23) reported 2/11 cases (18.18%) as echographic findings. In the present study, asymptomatic cases occurred in 25 (8.1%) patients, similar to those of other Mexican series, such as that of Velázquez et al. (24), who reported two (9%) cases. 

Multifocal disease was extremely rare in our study (0.6%), lower than that reported by Park et al. (28) with 9.6%, and by Jang et al. (29) with 3%. No bilateral tumors were identified. 

In Los Angeles County, Bernstein et al. (30) reported an increased incidence of PT in Latina Whites; Latin and Asian women were younger compared with general population. Women born in Mexico and Central and South America are at a 3 to 4 fold greater risk for developing malignant PT than Latina Whites born in the U.S. Pimiento et al. (15) identified that PT in Hispanic patients tend to have a higher rate of aggressive histopathological features, without racial differences in outcome at a median of 13 months of follow-up. Mastectomy was performed in 50% of Hispanic cases; more frequently compared with non-Hispanic patients, and re-excision was reported in 34% to obtain an at least 2-mm negative margin. In other Mexican series, mastectomies were carried out in 36.4 to 46.6% of patients (14,24). In our study, the number of mastectomies performed was lower (30.1%), and a second surgery was required in 12.7% to obtain >5 mm negative margins. Very small tumors are not very frequently reported in PT and could require the use of needle-guided excision for optimal surgical treatment, as occurred in four cases of our series. Oncoplastic procedures are frequently used in our hospital, were and applied in 23% of PT in patients who underwent conservative surgery. To our knowledge, this modality has not been registered in other Hispanic series. 

In published series, local recurrence was reported in 18.9 to 32% in a follow-up of 33 to 91 months, with median time after surgery of 17 months, and distant metastases occurs in 10 to 26% (27,29). In malignant PT, local and distant metastases were reported in 40 and 27%, at a median follow-up of 28 and 25.6 months, respectively (13). Global recurrence in Mexican series is reported in 13.6% after 73.4 months of follow-up (24). According to tumor subtype, recurrence in benign, borderline, and malignant PT is reported in 9.0, 37.5, and 53.3% of patients, respectively, with a follow-up of 44.7, 43.2, and 19.9 months, respectively (14). In the present study, after a mean verifiable follow-up of 36.2 months, corroborated recurrences occurred in 8.2% cases from the 254 patients with follow-up, fewer than in other series, the majority of these involving local relapse. Six (18.7%) patients with malignant PT developed recurrence; the majority of these were distant metastases.

## Conclusion

PT in this Mexican population had smaller tumors, more benign, and fewer malignant PT, with corroborated recurrences lower as compared with other Hispanic publications. Other characteristics are not very different from other Hispanic reports. Age increases with subtype severity. Malignant PT cases were older, postmenopausal, with less breastfeeding, and with more co-morbid stages such as systemic high blood pressure, diabetes, smoking, and higher BMI, compared with benign and borderline PT subtypes, with the majority of these differences statistically significant. 
